# Surgery for secondary hyperparathyroidism: mastering the anatomy – a single-center retrospective cohort study

**DOI:** 10.1186/s12882-026-04819-y

**Published:** 2026-02-28

**Authors:** Ali Murat Yildirim, Emre Kocabas, Banu Yilmaz, Gokalp Okut, Murat Karatas, Adam Uslu

**Affiliations:** 1 Clinic of General Surgery, University of Health Sciences Izmir City Hospital, Izmir, Turkey; 2 Clinic of Nephrology, Izmir City Hospital, Bornova, Izmir 35090 Turkey

**Keywords:** Secondary hyperparathyroidism, Chronic hemodialysis, Total parathyroidectomy, Parathyroid gland localization, Anatomical distribution, Bilateral cervical thymectomy, Surgical outcomes, Persistent hyperparathyroidism, Preoperative imaging, Endocrine surgery

## Abstract

**Background:**

This study aimed to evaluate the anatomical localization and distribution of parathyroid glands following total parathyroidectomy (TPTx) and bilateral cervical thymectomy (BCTx) in chronic hemodialysis patients with medically refractory secondary hyperparathyroidism (SHPT).

**Methods:**

A retrospective study on 154 consecutive SHPT patients with a mean hemodialysis duration of 109.3 ± 63.3 months was conducted.The study focused on the distribution of parathyroid glands within defined anatomical zones identified during surgery. Preoperative imaging methods (ultrasound and technetium-99 m sestamibi scintigraphy) provided limited diagnostic value, successfully localizing glands in only 34.6% and 31% of cases, respectively. Intraoperative parathyroid hormone (iPTH) measurement was not performed in any patient undergoing TPTx and BCTx.

**Results:**

Postoperative success was defined by the normalization of iPTH levels, with 76.6% of patients achieving normal iPTH levels on postoperative day one (mean: 12.2 ± 14.1 pg/mL). Persistent SHPT was identified in 36 patients, leading to five complementary parathyroidectomies. The study demonstrated that the anatomical zones defined for parathyroid gland localization are reliable, exhibiting higher accuracy compared to preoperative imaging.

**Conclusions:**

The findings support the feasibility of achieving high success rates with TPTx for SHPT, even when preoperative diagnostic tools are limited or ineffective. Knowledge of parathyroid gland distribution within these anatomical zones can significantly assist endocrine surgeons, particularly those who approach this condition with hesitation.

## Introduction

A linear correlation exists between dialysis duration and the requirement for surgery to manage secondary hyperparathyroidism (SHPT). While approximately 15% of patients on hemodialysis for 10 years necessitate parathyroidectomy, this rate escalates to nearly 40% in those with a 20-year dialysis history [1, 2]. Despite the substantial surgical need, current parathyroidectomy rates fall short of addressing this demand. According to the 2022 Turkish Society of Nephrology Registry, only 158 (2.0%) of 7,848 hemodialysis patients receiving medical treatment for SHPT underwent parathyroidectomy [3]. Similarly low rates are observed in the United States. Between 2004 and 2016, the parathyroidectomy rate for SHPT per 1,000 end-stage renal disease patients declined from 6.07 to 3.67 [4]. The introduction of calcimimetics and paricalcitol may account for this post-2005 decrease [5]. However, the reluctance of many endocrine surgeons in our country to operate on SHPT patients cannot be overlooked. The primary reason is the complexity and morbidity associated with total parathyroidectomy (4/4 parathyroidectomy), particularly in dialysis patients ineligible for transplantation. Indeed, series reports indicate that 72% to 98.5% of cases involve resection of all four glands [6, 7].

The aim of this study is to evaluate the distribution of parathyroid glands according to predefined anatomical zones and to assess the surgical outcomes of total parathyroidectomy (TPTx) combined with routine bilateral cervical thymectomy (BCTx) in patients with medically refractory SHPT. Specifically, we aimed to determine whether this standardized anatomical approach yields acceptable biochemical clearance without routine preoperative imaging or intraoperative PTH monitoring.

### Anatomical considerations

**Fig. 1:**
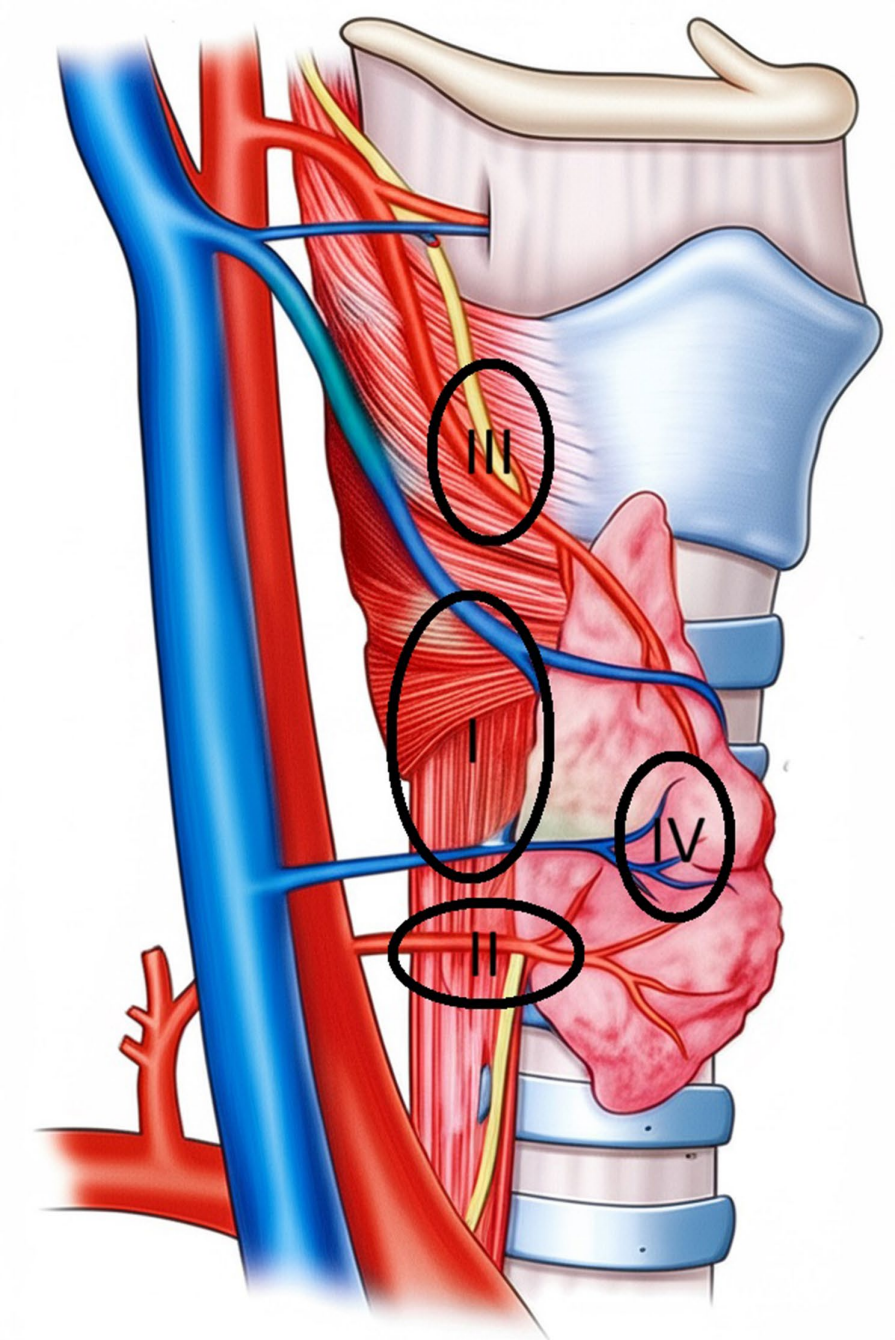
Anatomical sites of superior parathyroid glands. *Zone I: The area at the posterior aspect of the thyroid gland between the cricothyroid ligament and the anatomical demarcation of the recurrent laryngeal nerve and inferior thyroid artery. *Zone II: The area below the point where the inferior thyroid artery enters the thyroid gland and crosses the inferior laryngeal nerve. *Zone III: Cranially and along the posterior aspect of the superior thyroid artery. *Zone IV: Intrathyroidal

**Fig. 2:**
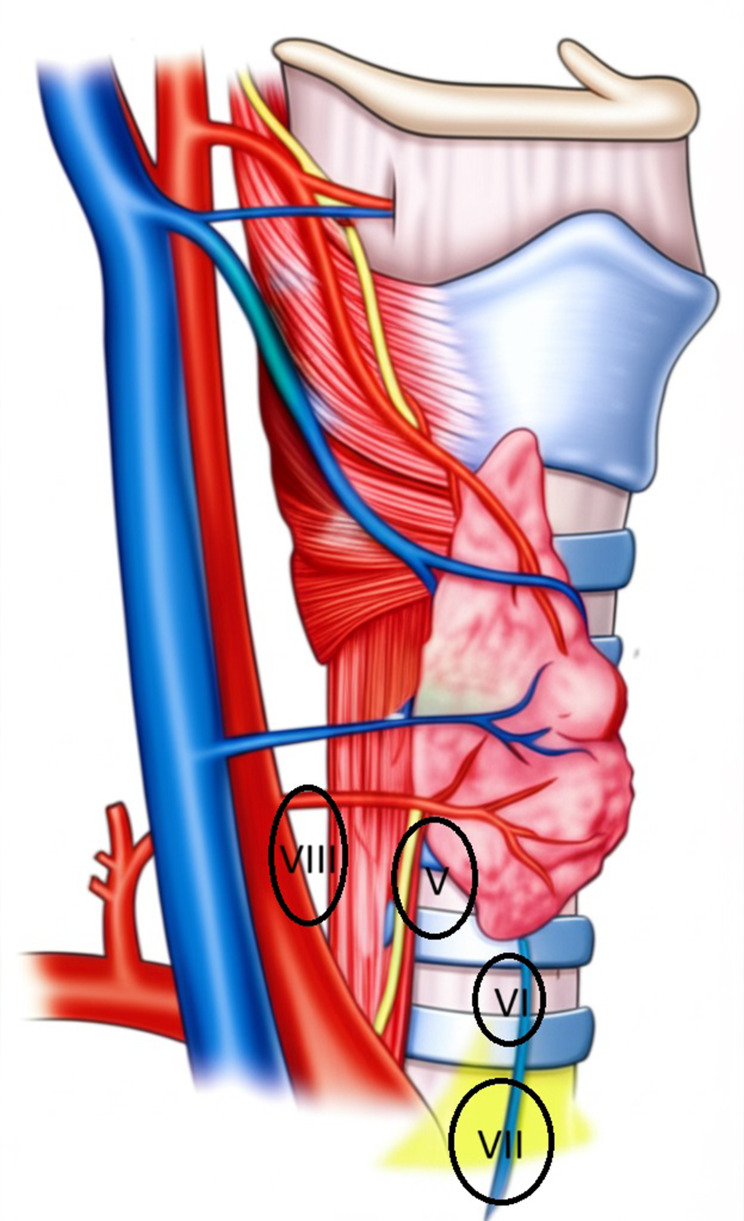
Anatomical sites of inferior parathyroid glands. *Zone V: At the posterior aspect of the thyroid gland between the junctions of the inferior thyroid artery/thyroid gland and the inferior thyroid vein/thyroid gland. *Zone VI: Within 1.0–1.5 cm caudally from the point where the inferior thyroid vein enters the thyroid gland. *Zone VII: Along the thyrothymic ligament. *Zone VIII: Between the carotid sheath and esophagus below the lower pole of the thyroid gland

The normal anatomical locations for the upper and lower parathyroid glands were previously defined and illustrated in Figs. 1 and 2 [8].

## Materials and methods

This single-center, retrospective cohort study expands upon our previous investigation, which reported the early outcomes and surgical protocol for the initial 60 patients in this cohort [8]. The study design, setting, and patient inclusion/exclusion criteria are detailed in this prior publication [8].

### Landis 4 criteria and reporting compliance

This study is a single-center, retrospective cohort analysis. As a result, the criteria of randomization and blinding were not applicable to the study design. Inclusion/Exclusion criteria are clearly defined in the “Study Population” section, based on patients failing to maintain adequate serum calcium, phosphate, and PTH levels despite maximized medical therapy, as per the 2009 KDIGO guidelines for CKD and mineral bone disorders. The sample size was determined by the consecutive number of medically refractory secondary hyperparathyroidism patients who underwent the defined surgical procedure between October 2013 and May 2024, and no formal power calculation was performed due to the nature of this retrospective case series.

### Study population

This study included 154 consecutive hemodialysis patients with SHPT who underwent initial parathyroidectomy between October 2013 and May 2024. Surgical indications were based on the 2009 KDIGO guidelines for CKD and mineral bone disorders [9]. As detailed previously [8], patients were referred for surgery if they failed to maintain adequate serum calcium (8.4–10.2 mg/dL), phosphate (2.5–4.6 mg/dL), and PTH levels (130–600 pg/mL) despite maximized medical therapy.

### Surgical procedure

Our standard surgical protocol, consistent with our previous report [8], was applied to all patients. This procedure consisted of total parathyroidectomy (TPTx) without autotransplantation and bilateral cervical thymectomy (BCTx), performed under general anesthesia via a Kocher neck incision. In this study, BCTx refers to the surgical en bloc excision of the complete thyrothymic ligament and bilateral superior thymus tissue. Surgical adequacy was defined by the pathological identification of at least 4 parathyroid glands and a postoperative day 1 (POD1) iPTH level within the normal range (< 60 pg/mL) [8]. Intact parathyroid hormone levels were quantified using the Access Intact PTH assay (Beckman Coulter, Inc., Fullerton, CA).

### Postoperative management

A standardized postoperative medical treatment protocol was implemented for all patients. This protocol included intravenous calcitriol (1 mcg twice daily) and 10% calcium gluconate infusion (1.0 mg elemental calcium/kg/hour) for 2–4 days. Oral calcium carbonate (3–6 g of elemental calcium per day) was administered as a supplement, along with vitamin D3 and magnesium as needed. The criteria for discharge included the resolution of symptoms, absence of QTc prolongation or arrhythmia on ECG, and corrected calcium levels exceeding 7.5 mg/dL.

### Preoperative imaging and intraoperative iPTH

In line with our prior protocol [8], most patients underwent preoperative cervical ultrasound and 99mTc-MIBI scintigraphy. However, these preoperative imaging results did not influence the standardized surgical approach (TPTx plus BCTx). Due to institutional constraints (National Social Security Institution limitations on monthly iPTH testing), intraoperative iPTH measurement was not performed in any patient [8]. Surgical success was therefore assessed using POD1 iPTH levels.

### Statistical analysis

All analyses were performed using IBM SPSS Statistics for Windows, Version 27.0 (IBM Corp., Armonk, NY). The normality of data distribution was assessed using the Kolmogorov-Smirnov test and visual inspection of histograms. Continuous variables with normal distribution were expressed as mean ± standard deviation (SD), while non-normally distributed variables (e.g., follow-up duration, PTH levels) were presented as median (minimum-maximum). Categorical variables were summarized as frequencies and percentages. A paired t-test was employed to compare preoperative and postoperative values for normally distributed data, whereas the Wilcoxon signed-rank test was used for non-normally distributed paired data. A *p*-value of < 0.05 was considered statistically significant (Table 1).

## Results

**Table 1:** The demographics and biochemistry profile of SHPT patients

Characteristic	Value
**Age (years)** ᵃ	49.1 ± 11.7
**Gender, n (%)**	
Male	88 (57.1%)
Female	66 (42.9%)
**Duration of hemodialysis (months)** ᵇ	87 (2–430)
**Preoperative Calcium (mg/dL)** ᵃ	11.6 ± 1.7
**Preoperative Phosphorus (mg/dL)** ᵃ	5.7 ± 1.68
**Ca x P product (mg²/dL²)** ᵃ	48.5 ± 17.9
**Preoperative iPTH (pg/mL)** ᵇ	1477 (115–3506)
**Postoperative Day 1**	
Calcium (mg/dL) ᵃ	8.6 ± 1.56
Phosphorus (mg/dL) ᵃ	3.8 ± 1.26
Intact PTH (pg/mL) ᵇ	11.5 (0–1087)
**Follow-up period (months)** ᵇ	12 (0–117)

ᵃValues are presented as Mean ± Standard Deviation (SD).

ᵇValues are presented as Median (Minimum – Maximum) due to non-normal distribution.

No. of patients: 154

### Patient characteristics

This study included 154 consecutive hemodialysis patients with SHPT who underwent TPTx & BCTx (Table 1). The mean age was 49.1 ± 11.7 years (range: 20–73 years) with a gender distribution of 88 (57.1%) males and 66 (42.9%) females. All patients were on long-term hemodialysis with a median duration of 87 months (range: 2–430 months**)**. Patients presented with very high preoperative iPTH levels (median: 1477 pg/mL; range: 115–3506 pg/mL) and large parathyroid glands observed intraoperatively. The initial phase of the study involved 60 patients, with results published in 2019. The overall median follow-up period was 12 months (range: 0–117 months).

### Parathyroid gland distribution and localization

The parathyroid glands and thyrothymic ligament tissue removed during surgery were sent for separate pathological analysis. Pathologists identified a total of 593 parathyroid glands. There were 27(%17.5) cases with 3 glands, 123(%79.9) patients with 4 glands, and 4(%2.6) patients with 5 glands identified. Table 2 details the number of glands removed and their distribution according to anatomical zones.

**Table 2:** The distribution of parathyroids by zone

Gland Location / Zone	Right Side	Left Side
	*n*(%)	*n*(%)
**Superior Glands**		
Zone I	92 (60.2)	84 (58.3)
Zone II	41 (26.8)	42 (29.2)
Zone III	12 (7.8)	13 (9.0)
Zone IV	8 (5.2)	5 (3.5)
**Inferior Glands**		
Zone V	103 (69.6)	98 (66.2)
Zone VI	15 (10.1)	17 (11.5)
Zone VII	18 (12.2)	22 (14.9)
Zone VIII	12 (8.1)	11 (7.4)

In 30 patients where right or left upper parathyroid glands were unclear, a simultaneous and unilateral thyroidectomy was performed during surgery. Pathological examination confirmed intrathyroidal localization (Zone IV) of the upper parathyroid gland in 13 of these cases. Thus, our study population demonstrated an 8.4% prevalence of upper parathyroid glands located within the thyroid gland itself.

Additionally, 13 patients underwent bilateral total thyroidectomy due to preoperative ultrasound findings suggestive of hypoechoic solid nodules with or without microcalcifications. Papillary thyroid carcinoma was identified in one patient, and papillary thyroid microcarcinoma was present in five of these cases.

### Preoperative imaging and parathyroid gland localization

Preoperative radiodiagnostic methods provided limited information in our study. Parathyroid ultrasonography successfully identified the dominant parathyroid gland and its clear location in only 52 patients (33.8%). Similarly, sestamibi scintigraphy was only able to visualize any single parathyroid gland in 46 cases (29.9%).

### Postoperative iPTH levels and surgical success

As shown in Table 3, postoperative iPTH values dropped to the normal range for 105 out of 123 patients (85.4%) who had 4 parathyroid glands removed. However, 18 cases exhibited persistent high iPTH levels (Median: 115.9 pg/mL). In seven of these cases, BCT was deemed inadequate, suggesting the likely presence of a missed fifth parathyroid gland or an ectopic focus. For the remaining 11 cases, the cause of persistence remained unclear. Biochemical cure was achieved in only 9 of the 27 patients (33.3%) who had three parathyroid glands removed. Overall, 118 patients (76.6%) exhibited normal iPTH levels on POD1. Importantly, no cases of recurrent secondary hyperparathyroidism were observed during the follow-up period. Although a mild increase in iPTH levels was noted at the last follow-up compared to the immediate postoperative period, values remained within acceptable clinical limits.

**Table 3:** Total number of parathyroid glands removed and corresponding postoperative intact PTH values

Group (No. of excised glands)	*n*	Postoperative (POD1) iPTH (pg/mL)Median (Minimum – Maximum)
**4 Glands Excised**	123	
Successful (Cure)	105	4.7 (0–53.0)
Persistent (Failure)	18	115.9 (73–1087)
**3 Glands Excised**	27	
Successful (Cure)	9	6.5 (0.2–46.0)
Persistent (Failure)	18	141.5 (70–483.1)
**5 Glands Excised**	4	8.5 (0–62.0)

Note: Data are presented as Median (Minimum – Maximum) due to non-normal distribution. Success was defined as a postoperative iPTH level < 65

### Management of persistent hyperparathyroidism

Persistent secondary hyperparathyroidism was identified in 36 patients on POD1 following TPTx+BCTx. Pathological examination of surgical specimens revealed 4 parathyroid glands in 18 patients and 3 parathyroids in the remaining 18 cases. Five of these 36 patients underwent early (within two days of TPTx+BCTx) completion parathyroidectomy. Since preoperative neck 99mTc-MIBI SPECT/CT failed to localize the missing parathyroid gland, the surgical exploration was guided by the anatomical location of the missing gland as deduced from the pathological analysis of the excised specimens.

## Discussion

In this study, we presented a series of 154 SHPT patients who underwent TPTx and BCTx, with complete follow-up data. Our previously defined surgical procedure and early results were published in 2019 [8]. The primary surgical success rate was 76.6%, Following early re-exploration in selected persistent cases, the cumulative surgical success rate reached 79.9%.

In our series, persistent elevation of iPTH was observed in 18 of the 123 patients (14.6%) despite the pathological confirmation of four excised glands. The median postoperative iPTH in this subgroup was 115.9 pg/mL, strongly suggesting the presence of a functional supernumerary gland. This finding aligns with the existing literature, which indicates that more than four parathyroid glands may be present in 13–19% of cases [10, 11].In our series, 18 patients exhibited persistent secondary hyperparathyroidism despite the excision of four glands, with a median iPTH level of 115.9 pg/mL (range: 73–1087 pg/mL). In 18 of these cases, calcimimetic therapy was discontinued. To avoid potential adynamic bone disease associated with excessively low iPTH levels (below 2 times the upper limit of normal) in SHPT patients [12], a second surgical intervention was deemed unnecessary, based on a consensus between the surgeon and nephrologist.

A recent study on total parathyroidectomy in patients with renal hyperparathyroidism classified PTX as total when iPTH fell below 10 pg/mL and subtotal when iPTH was between 10 and 65 pg/mL. Using these criteria, only 27 of 50 hemodialysis patients (54%) achieved biochemically total PTX, while 19 (38%) had subtotal PTX. Persistent secondary hyperparathyroidism was observed in four cases (8%) [13].

Our study underscores the challenge of achieving a complete parathyroidectomy. A key factor contributing to this difficulty is the potential for parathormone secretion from functional isolated cell nests within paratracheal and esophageal fat tissue, particularly under continued uremic stimulation [14, 15]. Another study involving 46 sHPT patients who underwent total PTX without autotransplantation revealed that four parathyroid glands were removed in 34 patients (74%), while fewer than four glands were identified in 6 patients (13%), leading to persistent sHPT [16]. This observation mirrors our findings, where the median postoperative iPTH levels were markedly higher in patients with incomplete resection (two or three glands) compared to those in whom all four glands were excised.

A recent systematic analysis of the prevalence and location of parathyroid glands in hyperparathyroidism patients examined 14 intraoperative studies (*n* = 3399 patients). This analysis indicated that 82.2% of patients had four glands [11]. A large anatomical study demonstrated that the upper parathyroid glands are typically located posterior to the cricothyroid junction (77%) and less commonly posterior to the upper pole (22%) [17]. These locations correspond to Zones I and II, as defined in our study. We and other investigators have emphasized that the intersection between the laryngeal nerve and the lower thyroid artery serves as a boundary, with the upper parathyroids almost always situated above this plane [18]. Regarding the lower parathyroids, they are commonly found on the anterior or latero-posterior surface of the lower thyroid pole (42%) or on the thymic tongue (39%). These locations align with our Zones V, VI, and VII [17, 19].

While not previously reported, a recent small anatomical study identified ectopic parathyroid glands within the thyroid parenchyma (Zone IV) at a low rate of 5.4% [20]. In our series, 13 patients (8.4%) had upper parathyroid glands located within the thyroid gland.

A study conducted by Numano et al. between 1981 and 1996 on 570 patients with secondary hyperparathyroidism revealed that 1% of cases had 3 parathyroid glands after the first operation, while 82.8% had 4 glands and 14.6% had 5 glands after the second operation [6]. The anatomical locations of the glands in 92 patients with multiple parathyroid glands were consistent with the zones we described, specifically Zones II-IV-V-VI, and VIII.

In our study, intraoperative iPTH analysis was not feasible, and preoperative radiodiagnostic methods proved to be of limited value.

The current guidelines of the European Association of Nuclear Medicine (EANM) recommend using [99 m Tc]Tc-MIBI SPECT/(CT), 4D-CT, or positron emission tomography (PET) using the radioactive tracer [18 F]-fluorocholine in conjunction with preoperative ultrasonography in cases of primary hyperparathyroidism scheduled for parathyroid surgery. However, a widely accepted algorithm with high evidential value for preoperative radiological evaluation in cases of secondary hyperparathyroidism is yet to be available [21]. 

However, in 52 patients who underwent surgery due to (SHPT), the sensitivity and specificity rates for MR, 4D-CT, and US to detect the localisation of the parathyroid glands were 91%-89%, 66%-58%, and 64%-50%, respectively. The positive predictive value of MR plus 4D-CT reached 96.5% prior to surgery, suggesting that this combined method may be very useful in cases where the parathyroid glands cannot be clearly isolated [22]. Nevertheless, considering the high cost and limited accessibility of these advanced imaging modalities in many institutions, BCT remains a practical and cost-effective surgical strategy to ensure high cure rates.

Similarly, in 25 patients diagnosed with secondary hyperparathyroidism, the combined use of ultrasonography and 4D-CT provided the highest diagnostic performance (sensitivity 1.000, accuracy 0.989), and the use of this combined method was emphasized to achieve optimal preoperative localization [23]. 

In light of these recent publications, routine 4D CT scanning appears to be a rational approach to detect abnormal location of the parathyroid gland in the mediastinum or neck and to improve surgical success rates. However, considering the limited availability and high cost of 4D-CT in many clinical settings, performing Bilateral Cervical Thymectomy (BCT) as a standard adjunct to TPTx serves as a crucial surgical strategy to ensure the removal of potential ectopic glands that preoperative imaging might otherwise miss or when such advanced imaging is inaccessible. We achieved a success rate of nearly 80% in total parathyroidectomy by adhering to the described anatomical zones. To our knowledge, this is the most informative mapping of parathyroid gland location to date. Therefore, even in the absence or inadequacy of complementary diagnostic methods, it is highly possible to identify parathyroid glands by relying solely on the defined zones. We believe that the anatomical zones we have described will be of significant practical benefit in managing secondary hyperparathyroidism, a condition that many endocrine surgeons approach with hesitation.

### Study limitations

This study has several limitations that should be considered. Firstly, it is a single-center, retrospective cohort study, which limits the generalizability of the findings and is subject to potential institutional bias. Secondly, intraoperative intact PTH (iPTH) measurement was not performed in our cohort. Although iPTH is considered the gold standard for primary hyperparathyroidism, its routine necessity in secondary hyperparathyroidism remains debated, and our protocol relied on postoperative day one iPTH values.While the anatomical mapping achieved a high success rate, the lack of intraoperative confirmation means the immediate success could not be validated in real-time. Thirdly, although recent literature suggests that routine supplementary 4D CT scans could enhance the detection of ectopic glands, limitations in accessibility and cost prevented their use in our series.Finally, as a retrospective series, the determination of the sample size was based on consecutive patients undergoing surgery and did not involve a formal power calculation.

## Conclusion

Total parathyroidectomy combined with bilateral cervical thymectomy constitutes a safe and definitive treatment strategy for secondary hyperparathyroidism. Our findings demonstrate that adhering to a systematic anatomical roadmap—specifically the described parathyroid zones—yields high biochemical cure rates even in the absence of advanced preoperative imaging or intraoperative parathormone monitoring. Given the significant prevalence of supernumerary glands and ectopic foci observed in our series, the routine addition of BCT is strongly justified to minimize the risk of persistence. This standardized approach offers a practical, replicable, and cost-effective solution for surgeons managing this complex patient population.

## Data Availability

The datasets generated and/or analysed during the current study are available from the corresponding author on reasonable request.

## Abbreviations

BCTx: Bilateral cervical thymectomy
SHPT: Secondary hyperparathyroidism
TPTx: Total parathyroidectomy
iPTH: Intraoperative/Intact Parathyroid Hormone
99mTc-MIBI: Technetium-99 m sestamibi
KDIGO: Kidney Disease: Improving Global Outcomes
CKD-MBD: Chronic Kidney Disease-Mineral and Bone Disorder
POD1: Postoperative day 1
SD: Standard deviation
CI: Confidence Interval
